# Physicochemical Properties of Cellulose-Based Hydrogel for Biomedical Applications

**DOI:** 10.3390/polym14214669

**Published:** 2022-11-02

**Authors:** Sreeja Harikumar Aswathy, Uttamchand NarendraKumar, Inderchand Manjubala

**Affiliations:** 1 Department of Biosciences, School of Biosciences and Technology, Vellore Institute of Technology (VIT), Vellore 632014, India; 2 Department of Manufacturing, School of Mechanical Engineering, Vellore Institute of Technology (VIT), Vellore 632014, India

**Keywords:** hydrogels, carboxymethyl cellulose, crosslinking, swelling, biomedical, tissue engineering

## Abstract

Hydrogels are three-dimensional network structures of hydrophilic polymers, which have the capacity to take up an enormous amount of fluid/water. Carboxymethyl cellulose (CMC) is a commercially available cellulose derivative that can be used for biomedical applications due to its biocompatibility. It has been used as a major component to fabricate hydrogels because of its superabsorbent nature. In this study, we developed carboxylic acid crosslinked carboxymethyl cellulose hydrogels for biomedical applications. The physicochemical, morphological, and thermal properties were analyzed to confirm the crosslinking of carboxymethyl cellulose. Fourier-transform infrared spectra confirmed the crosslinking of carboxymethyl cellulose with the presence of peaks due to an esterification reaction. The distinct peak at 1718 cm^−1^ in hydrogel samples is due to the carbonyl group vibrations of the ester bond from the crosslinking reaction. The total carboxyl content of the sample was measured with crosslinker immersion time. The swelling of crosslinked hydrogels showed an excellent swelling capacity for CG02 that is much higher than CG01 in water and PBS. Morphological analysis of the hydrogel showed it has a rough surface. The thermal degradation of hydrogel showed stability with respect to temperature. However, the mechanical analysis showed that CG01 has a higher compressive strength than CG01. The optimum swelling ratio and higher compressive strength of CG01 hydrogels could give them the ability to be used in load-bearing tissue regeneration. These results inferred that the carboxylic acid crosslinked CMC hydrogels could be a suitable matrix for biomedical or tissue-engineering applications with improved stability.

## 1. Introduction

The main difficulty facing the biomedical sector is producing bioactive materials with optimal characteristics for tissue engineering applications. Hydrogels have recently attracted significant interest as potential candidates for biological applications because of their similarity to the natural extracellular matrix. Due to the hydrophilic functional groups, hydrophilic materials such as hydrogels can absorb a significant amount of water and develop a 3D network structure [1,2]. The properties like biocompatibility, biodegradability and less immunogenicity are the advantages of hydrogels over other biomaterials [3]. Hydrogels are made from both synthetic and natural polymers. Synthetic polymers include polyacrylic acid, polyvinyl alcohol, polyphosphazene, etc., and naturally derived polymers include collagen, chitosan, agarose, hyaluronate, cellulose derivatives, etc. [4]. Amongst the naturally derived polymers, chitosan is a well-studied polysaccharide biopolymer for biomedical applications [5]. Generally speaking, naturally derived polymers are preferred over synthetic polymers because they are more plentiful, renewable, biodegradable, and produce more biocompatible degradation products. Because of their poor crosslinking, biopolymers have very little strength and stability in water and under physiological conditions. The two types of crosslinking reactions that strengthen and stabilize hydrogels are physical crosslinking, also known as physical gels, and chemical crosslinking, also known as chemical gels. Chemical gels are irreversible gels produced by chemical crosslinks by covalent interactions, whereas physical gels are reversible gels produced by hydrogen bonds or hydrophobic interactions [6]. A recent study reported the synthesis of hemicellulose-based Arabinoxylan hydrogel, which is chemically crosslinked with polyvinyl alcohol via TEOS as a crosslinker [7].

Cellulose is the major structural polysaccharide found in the plant cell wall and is also produced by some microorganisms. The glucose units are joined by β-glycosidic linkages to form cellulose polymer [8]. Since the 19th century, cellulose—one of the most prevalent polymers on earth—has been used in various products like gels, film, and viscosifiers [9]. Because of their biocompatibility, cellulose and cellulose derivatives are now used in biomedical and pharmaceutical applications. Cellulosic materials have the important advantage that their chemical structure can be easily changed through functionalization. Based on the chemical process, cellulose derivatives are divided into cellulose ether and cellulose ester. Cellulose ether derivatives include methyl cellulose, ethyl cellulose, hydroxyethyl cellulose, carboxymethyl cellulose, and hydroxypropyl cellulose, while cellulose ester derivatives include cellulose acetate, cellulose nitrate, and cellulose sulfate [10]. Carboxymethyl cellulose (CMC) is the major commercially available cellulose ether derivative. It is considered the gold standard cellulose derivative because of its polyelectrolyte nature where it is sensitive to pH and ionic variations [11]. CMC is a water-soluble cellulose derivative made by attaching carboxymethyl groups to anhydro-glucose units in a chemical reaction with chloroacetic acid [12]. It is an anionic polysaccharide [13] and a superabsorbent polymer with an excellent swelling capacity. The swelling capability is due to the electrostatic charges of the polymer network. However, it is less stable in fluid/water due to its highly hydrophilic nature [14]. Crosslinkers can be utilized to overcome the lack of mechanical strength and stability. Epichlorohydrin, urea derivatives, aldehyde derivatives, carboxylic acids, etc., can be used as crosslinking agents for cellulose-based hydrogels [15]. The primary goal of this research is to create a crosslinked CMC hydrogel in a form that is more stable in physiological conditions. Our group has developed a CMC membrane for full-thickness healing of normal and diabetic wounds and a citric acid crosslinked scaffold for bone regeneration [16,17]. Additionally, Capanema et al., reported that they have developed a CMC-based superabsorbent hydrogel for wound healing applications [18]. The main objective of this study is to develop a crosslinked CMC hydrogel with improved stability in physiological conditions and its characterizations. In this study, cellulose-based hydrogel samples are prepared using the crosslinker immersion method, followed by drying. The CMC hydrogels are fabricated by both the immersion and drying processes, and the impact of equilibration is investigated. Equilibration of hydrogels is conducted in distilled water after immersion in crosslinking solution to understand the better crosslinking reaction compared with non-equilibrated hydrogels.

## 2. Materials and Methods

### 2.1. Fabrication of Cellulose Hydrogel

Hydrogels were prepared by dissolving sodium CMC (7% *w*/*v*) of viscosity (1500–3000 cP) (HiMedia, Mumbai, India) in distilled water (DW), molded into shapes, and immersed in 1 M citric acid (HiMedia, Mumbai, India) solution for different ranges of time varying from 1 h to 24 h. The hydrogels were washed with distilled water three times to remove the unreacted crosslinker residues. Some of the obtained hydrogels were vacuum dried at 45 °C for 48 h and named CG01. The remaining hydrogels were equilibrated with water for 24 h and then vacuum dried and named CG02. The dried hydrogels were stored in the refrigerator for analysis.

### 2.2. Fourier Transform Infrared Spectroscopy (FTIR)

The crosslinking of the carboxymethyl cellulose hydrogel in a dry state was confirmed using FTIR-Attenuated Total Reflectance (ATR) (Shimadzu Scientific Instruments, Kyoto, Japan). Spectra were recorded from 4000 cm^−1^ to 500 cm^−1^.

### 2.3. Carboxyl Content

The carboxyl groups present in the hydrogels were measured by a previously reported acid-base titration method [19]. Briefly, 0.1 g of vacuum-dried cellulose hydrogel was dispersed in 0.1 N NaOH and maintained with continuous stirring to allow its complete dispersion. The dispersion was then titrated against 0.1 N HCl using a phenolphthalein indicator. The total number of carboxyl groups present in the hydrogel was measured using Equation (1).

(1) $$\mathrm{Carboxyl}\;\mathrm{content}\;\left(\frac{\mathrm{mEq}}{100\;g}\right)=\left(\frac{\left(\mathrm{Va}-\mathrm{Vp}\right)\times \;N}{W}\right)\times 100$$

where Va and Vp are the volume of 0.1 N HCl in the absence and presence of hydrogel, N—normality of HCl, and W—the weight of the hydrogel. The total carboxyl content is mentioned here as xx mEq/100 g hydrogel.

### 2.4. Swelling Study

The swelling ability of hydrogels was evaluated in water and phosphate-buffered saline (PBS) (HiMedia, India) at predetermined time intervals of 3, 12, 24, and 48 h of immersion. The swelling ratio (%) was measured using Equation (2).

(2) Swelling ratio (%)=((Ws−Wi)/Wi)×100

where Ws is the weight of swollen hydrogel and Wi is the initial weight of the hydrogel.

### 2.5. Morphology of Hydrogel

The hydrogel was dehydrated using the different gradients of ethanol (50, 70, 80, and 90%) and acetone and then sputter-coated with gold–palladium using a mini sputter coater (SC7620, Quorum Technologies, Laughton, UK) and followed by scanning electron microscope imaging (Carl Zeiss, Oberkochen, Germany).

### 2.6. Thermal Analysis

The thermal stability of the hydrogels was determined by a thermogravimetric analyzer (TA Instruments, New Castle, DE, USA). The samples were heated within the range of 30 °C to 800 °C under nitrogen gas at a rate of 10 °C/min. The weight loss of the hydrogel was recorded and plotted against temperature.

### 2.7. Mechanical Study

The compression behavior of hydrogels was analyzed using a CT3 Texture analyzer (Brookfield AMETEK Inc., Middleboro, MA, USA) with a crosshead speed of 0.1 mm/s and trigger load of 5 g. The hydrogels of diameter 6 mm diameter and 8 mm height for dry and 8 mm diameter and 10 mm height for wet samples were compressed to 70% deformation. The prepared hydrogels were immersed in PBS for 15–30 min for wet measurements. The stress and strain values were calculated from the obtained data and the stress-strain graph was plotted using OriginPro 8.5.

### 2.8. Statistical Analysis

GraphPad Prism 9.3.1 was used to perform the statistical analysis of the obtained data. All the values presented here are expressed as Mean ± Standard deviation. The two-way ANOVA has been used with multiple comparisons test (*p* value; * < 0.05, ** < 0.01, *** < 0.001 and **** <0.0001).

## 3. Results and Discussion

### 3.1. Fabrication of Cellulose Hydrogel

**Figure 1:**
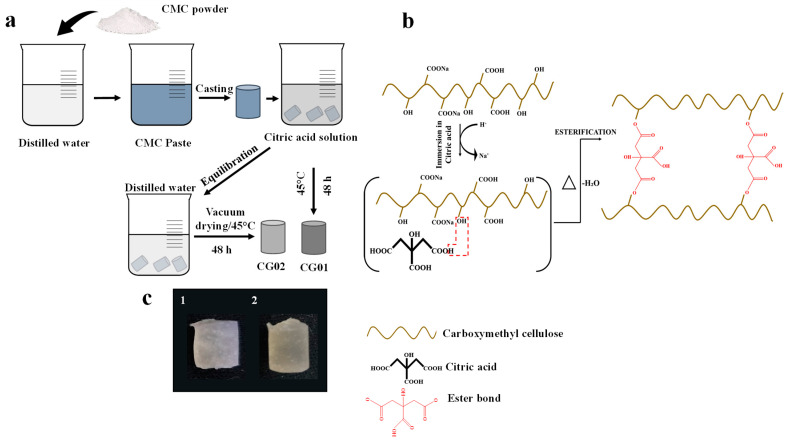
(**a**) Graphical illustration describes the method of preparation of CG01 and CG02 hydrogels, (**b**) crosslinking mechanism of hydrogels, and (**c**) photographic images of fabricated hydrogels CG01 (**c1**) and CG02 (**c2**).

The hydrogels were successfully fabricated via citric acid immersion and the vacuum drying method, and the fabrication process is shown in Figure 1a. The hydrogels prepared with and without equilibration, named CG02 and CG01, respectively, are shown in Figure 1c. During immersion in citric acid, the sodium ions present in the CMC backbone were replaced by hydrogen ions from the citric acid solution. This led to the diffusion of citric acid into CMC, resulting in hydrogel formation [20]. Citric-acid-mediated chemical crosslinking of CMC occurred via the esterification reaction during the vacuum-drying process. During crosslinking, the citric acid was converted to cyclic anhydride and esterified the hydroxyl groups of CMC for ester crosslinkages [21]. This crosslinking mechanism is shown in Figure 1b.

### 3.2. Fourier Transform Infrared Spectroscopy

**Figure 2:**
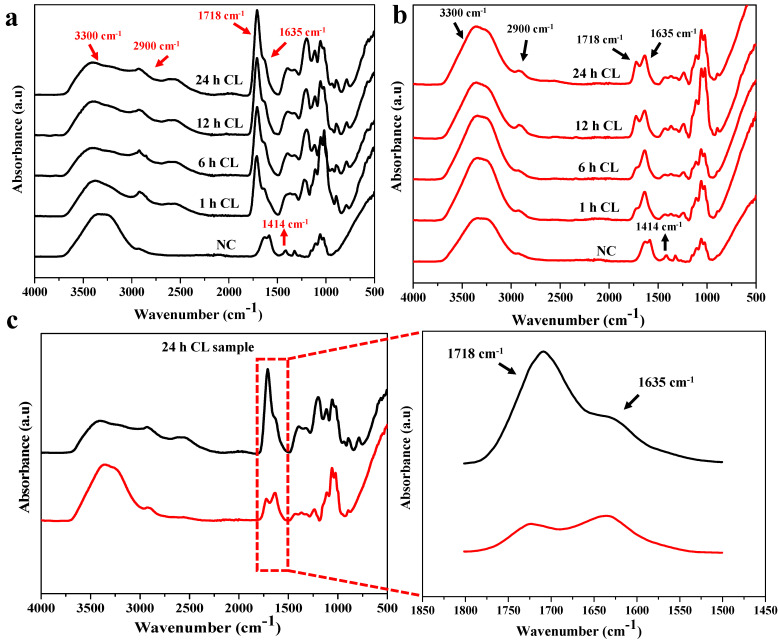
FTIR spectra of CG01 (**a**) and CG02 (**b**) hydrogel based on varying immersion times in crosslinker solution, and (**c**) compositional spectral analysis of 24 h crosslinked samples of both CG01(black line) and CG02 (red line).

Crosslinking of hydrogels was confirmed using Fourier transform infrared spectroscopy. Figure 2 shows the FTIR spectra of CG01 and CG02 hydrogels with different immersion times in citric acid solution for crosslinking reactions. The -OH stretching of CMC was observed at the range of 3300 cm^−1^ and the -CH stretching was noticed at around 2900 cm^−1^, for all the hydrogel samples. The absorption peaks at 1635 cm^−1^ and 1414 cm^−1^ were observed due to asymmetric and symmetric stretching of carboxylate groups (-COO-) in all samples [22,23]. Further, the peak intensity was reduced during the addition of citric acid, intending the formation of ester bonds with the OH groups. The intensity of the peak corresponding to the ester group positioned at 1718 cm^−1^ in CG01 and CG02, becoming higher over increasing crosslinking time, indicated the crosslinking reaction.

However, compared with CG02, the ester bond peak intensity was higher in CG01. The carbonyl peak intensity was higher in the 24 h crosslinked sample. The detailed spectral analysis of the 24 h crosslinked samples in both CG01 and CG02 is depicted in Figure 2c and highlights the esterification peak. The ester bond peaked at 1718 cm^−1^ of CG01 and CG02 confirmed the crosslinking reaction. However, a higher intensity of the ester bond peak was observed in CG01 compared to CG02. The absorption peak at 1635 cm^−1^ showed that all carboxylate groups were not participating in the esterification reaction. The distinct peak at around 1300–1200 cm^−1^ due to the C–O stretch of citric acid was seen in CG01 and CG02, but the intensity was more in CG01 due to the presence of a large amount of citric acid content. The—C–O–C stretching of the polysaccharide backbone was observed around 1065–1025 cm^−1^ in both the samples; where the peak intensity was low in CG01, it may have been because of the participation of more polymer chains in the esterification reaction. Likewise, the glycosidic linkage between glucose units in the carboxymethyl cellulose polymer was observed around 890 cm^−1^.

The esterification peak value of our study was comparable to that reported by Priya et al., where the characteristic band at 1721 cm^−1^ was assigned to the C=O vibrations owing to the esterification reaction of citric acid crosslinked CMC scaffold [22]. Additionally, Raucci et al. reported that the carbonyl peak at 1723 cm^−1^ was formed after citric acid treatment in cellulose-based hydrogel [24].

### 3.3. Total Carboxyl Content

**Figure 3:**
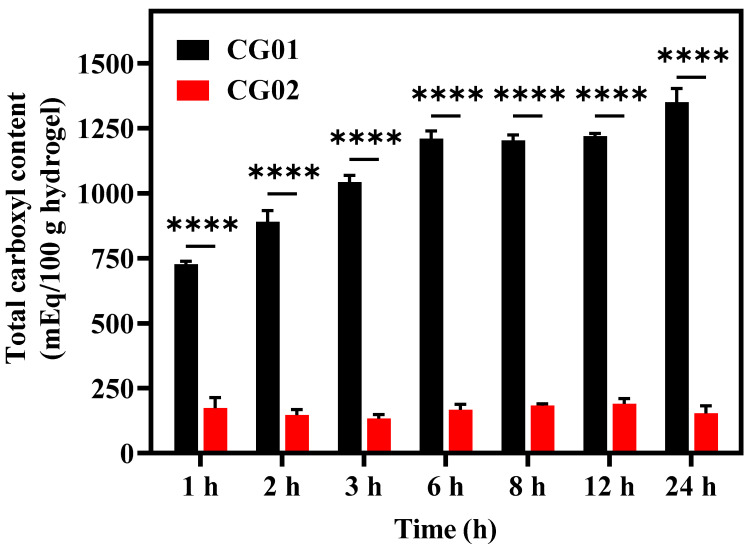
Total carboxyl content of CG01 and CG02 for different immersion times in crosslinker solution at room temperature. The results *p* < 0.0001 (****) were considered as significant in compared to CG02.

The degree of crosslinking of the cellulose polymer network in hydrogels can be determined by estimating the carboxyl content present in the hydrogel. This method measured the esterified and free carboxyl groups of citric acid [25]. The total carboxyl content of the hydrogel was determined for different time intervals of crosslinking reactions, ranging from 1 h to 24 h, and it was observed in the range of 726 to 1350 mEq/100 g and 133 to 190 mEq/100 g for CG01 and CG02 samples, respectively (Table 1) and illustrated in Figure 3. When the crosslinking time in citric acid increased, it was expected to have more crosslinking due to the higher esterification process. As seen in CG01, with an increase in time, the total carboxyl content increased from 726 mEq/100 g to 1350 mEq/100 g, indicating the value was reached twice within 24 h. In CG01, the ionic reaction and crosslinking were initialized immediately when it was immersed in a citric acid solution, which is why there was only a gradual increase in carboxyl content after 6 h. When the same samples were equilibrated in distilled water for 24 h, the total carboxyl content decreased from 726 mEq/100 g to 173 mEq/100 g of 1 h crosslinked samples and the carboxyl content did not change significantly over the time of 24 h of crosslinking in citric acid solution. There was no significant difference in the carboxyl content of CG02 as crosslinking time increased. This indicated that during equilibration for 24 h the unreacted citric acid residues were removed from the hydrogels. Therefore, by comparing both CG01 and CG02 we selected 24 h of citric acid-immersed samples for further studies.

### 3.4. Swelling Study

**Figure 4:**
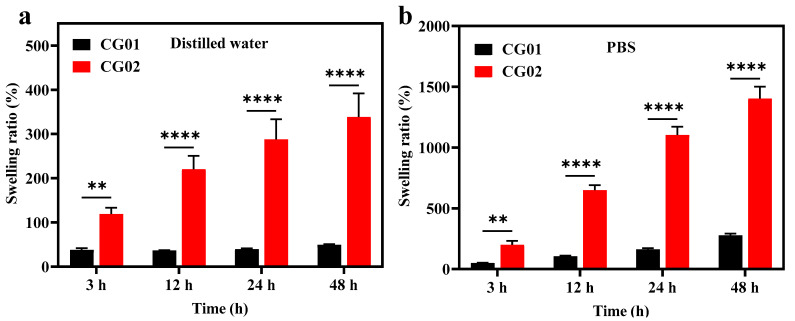
The swelling ratio of CG01 and CG02 samples after 3, 12, 24, and 48 h in (**a**) distilled water, and (**b**) PBS at 37 °C. The results *p* < 0.01 (**) and *p* < 0.0001 (****) were considered as significant in compared to CG02.

To study the effect of the swelling of hydrogels, the crosslinked hydrogel was immersed in distilled water and PBS (pH-7.4) at 37 °C for the predetermined time. Figure 4 shows the swelling ratio of CG01 and CG02 samples for 3, 12, 24, and 48 h. The rate of absorption for CG01 and CG02 increased with time in both PBS and water. The swelling ratio was higher in PBS compared to distilled water. As CMC is ionic sensitive, the carboxylate groups in the hydrogels were ionized by the ions present in the PBS solution, thereby increasing the electrostatic repulsion, which resulted in a high swelling ratio [26]. The CG02 samples showed an increased swelling ratio than CG01. The lower swelling rate in CG01 may be due to the high degree of crosslinking as a large number of citric acid residues were present in it. The higher swelling rate in CG02 was due to the presence of more free numbers of carboxylate groups, where most of the loosely crosslinked citric acid residues were removed during equilibration. The increase in osmotic pressure by the hydrophilic group present in the hydrogel and the dissociated sodium carboxylate were responsible for the swelling of hydrogels. It was also shown that the negative charge repulsion influenced the swelling of the hydrogels [27]. In the tissue engineering field, the proper swelling of hydrogels helps in the diffusion of nutrients and other molecules, which will further help in cell migration through the hydrogels [28]. Similarly, the swelling of hydrogels also plays an important role in drug delivery, where the swelling of hydrogels helps in the sustained release of drugs. Liu et al. fabricated rapid swelling and long-term gastric retention hydrogels based on polyacrylic acid and polyvinyl alcohol [29].

### 3.5. Morphology of Hydrogel

**Figure 5:**
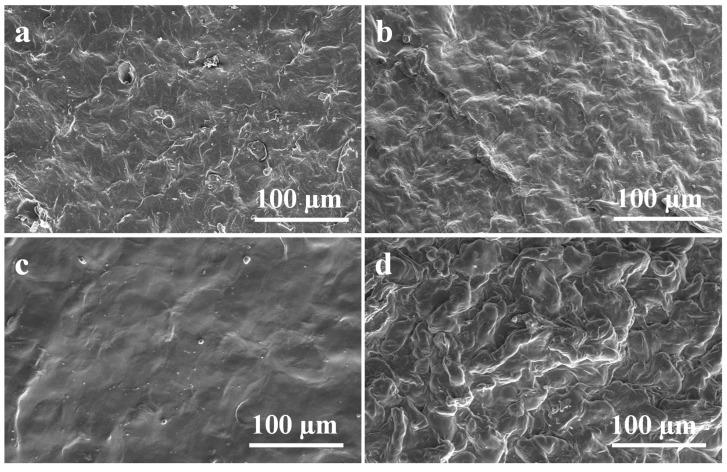
SEM images of surface morphology of dry (**a**) CG01, (**b**) CG02, and swollen (**c**) CG01, (**d**) CG02 hydrogels after immersion in PBS for 48 h.

The morphological feature of hydrogels was studied using a scanning electron microscope (SEM). Figure 5 illustrates the SEM image of CG01 and CG02 samples in the dry state and swollen CG01 and CG02 samples in PBS for 48 h. From the image, we can confirm that the hydrogel had a rough surface. The SEM image also confirmed that after 48 h of swelling in PBS there were no structural changes and the hydrogel did not lose its integrity. Trattnig et al. reported that a non-porous hydrogel scaffold was used for cartilage repair where the tissue was repaired by hydrogel degradation over time [30].

### 3.6. Thermal Analysis

**Figure 6:**
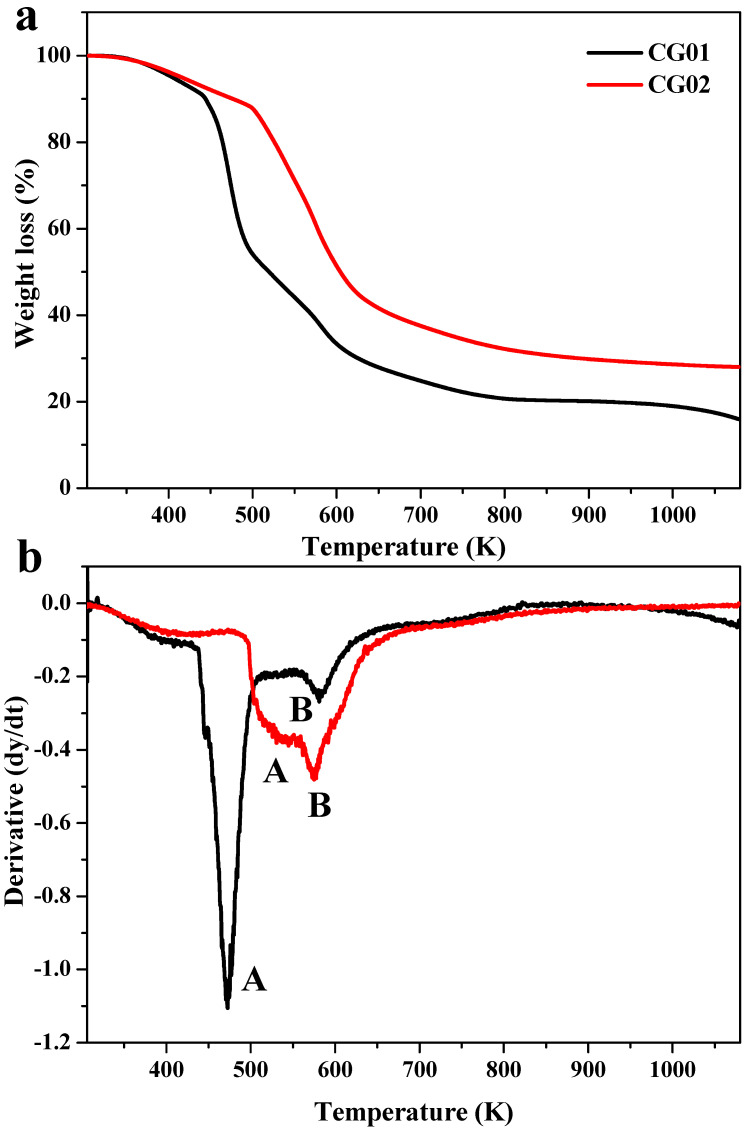
Thermogravimetric analysis of CG01 and CG02 hydrogels (**a**) TGA curve, and (**b**) its first derivative showing clearly the difference in the peak at 470 K.

The thermogravimetry analysis (TGA) gives the relationship of the material’s stability to temperature. Figure 6a,b shows the thermal curves for CG01 and CG02 samples and their derivatives, respectively. The thermal degradation temperature, % weight loss, and residual content are specified in Table 2. The TGA thermogram of both samples showed major degradation in a two-step process. In CG01 and CG02, a small degradation region at 361 K was associated with the elimination of water from the hydrogels [31]. The degradation regions at 581 K and 576 K were a result of the decomposition of the polymeric backbone or degradation of ester bonds [25]. In CG01, the degradation at 472 K was because of the degradation of crosslinked citric acid residues, but in CG02 the degradation started at 532 K. The degradation of crosslinked residues of equilibrated hydrogels was increased by 60 K due to the smaller amount of citric acid residues. In a previous study, it was reported that the degradation of citric acid started at 433 K, and in hydrogel film, the degradation started at 472 K [21]. Our results concurred with the previous study, in that the decomposition of the crosslinked polymer backbone of the CG01 sample at 472 K was due to the high crosslinking density. As compared to CG02, the weight loss was higher in CG01 because in CG01 there were only a few free CMC chains. From this, we were able to conclude that both the hydrogels were thermally stable.

### 3.7. Mechanical Test

**Figure 7:**
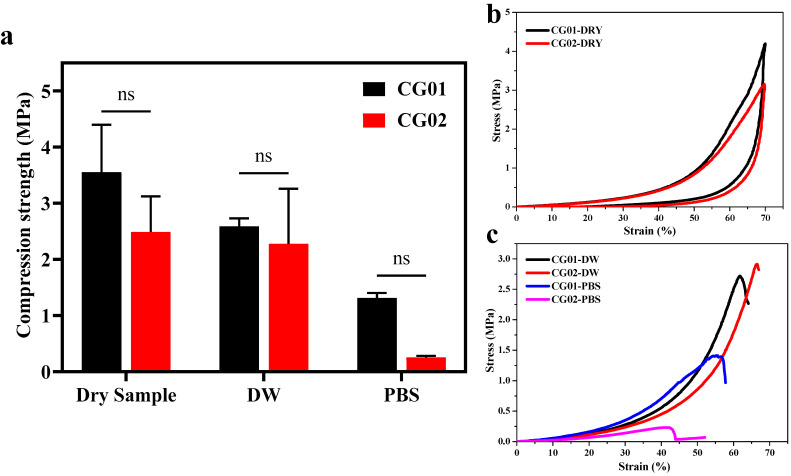
The compressive test analysis of hydrogels in wet and dry conditions (the samples were immersed in water or PBS for wet samples (**a**) compressive strength of CG01 and CG02 hydrogels at different conditions, and (**b**) stress-strain behavior of dry samples, (**c**) stress-strain curve of samples in wet conditions. (DW—Distilled water, PBS—Phosphate-buffered saline). The results are non-significant (ns) in compared to CG02.

The mechanical properties of the hydrogels were determined by a compression test, allowing the samples to be compressed to 70% strain (Table 3). Figure 7a shows the compressive strength of hydrogels CG01 and CG02 in dry conditions and wet conditions (distilled water and PBS). In dry conditions, the compressive strength of CG01 was 3.55 ± 0.84 MPa and for CG02 it was 2.48 ± 0.63 MPa. The decrease in compressive strength of CG02 hydrogels may be due to a lower crosslinking density than CG01. From the stress-strain graph, both CG01 and CG02 had a hysteresis curve, which represents the resilient behavior of hydrogel in dry conditions.

Mechanical stability is essential if material is utilized in biomedical applications, especially in the tissue engineering field, and therefore it is vital to test the compressive strength in wet conditions, mainly in PBS. The compressive strength of CG01 was 2.57 ± 0.20 MPa in water and 1.3 ± 0.09 MPa in PBS and for CG02 the compressive strength was 2.27 ± 0.98 MPa in water and 0.25 ± 0.03 MPa in PBS. The compressive strength of CG02 was lower than CG01 in both water and PBS. Nevertheless, in water the compressive strength of CG01 and CG02 hydrogels was more or less equal, but in PBS the compressive strength of CG02 was considerably lower than CG01 hydrogels. From the stress-strain graph, we found that a fracture occurred in the hydrogel after some strain. The stress-strain behavior of CG01 and CG02 in dry and wet conditions is illustrated in Figure 7b,c, respectively. It is to be noted that both samples showed recovery of stress in dry conditions. While the maximum stress held by CG01 was 3.55 ± 0.84 MPa, higher than CG02, the maximum stress was obtained at the given 70% strain. While in the wet conditions, when the samples were soaked in distilled water and PBS, the samples broke even at the 60% strain. The maximum stress was 2.57 ± 0.20 MPa, which was less than dry samples. The calculated compression strength was higher in CG01 than in CG02 in all conditions. The results suggested that the equilibration of hydrogels reduced the compressive strength of the hydrogels in PBS. Zheng et al. reported that the mechanical strength of the equilibrated carboxymethyl cellulose hydrogel was lower than the prepared hydrogels when immersion time in crosslinker solution ranged from 3 to 12 h [20]. In the biomedical or tissue engineering field, the mechanical property of a scaffold plays an important role because it should be more or less equivalent to the mechanical strength of the corresponding tissue to be reconstructed.

## 4. Conclusions

This study demonstrated the effect of the equilibration of crosslinked CMC hydrogel with tailored mechanical properties. The carboxylic acid crosslinked hydrogels with and without equilibration were successfully prepared. The ATR-FTIR results confirmed the formation of ester linkages and confirmed the crosslinking reaction. The duration of immersion of carboxymethyl cellulose hydrogels in citric acid solution was fixed to 24 h for a crosslinking reaction. The SEM images showed that the hydrogels were non-porous in structure and maintained their integrity after 48 h of swelling in PBS. The hydrogels showed good swelling ability in both distilled water and PBS. The ratio of the hydrogel swelling was more in PBS than in distilled water. In both solutions, as time increased, the swelling ratio of hydrogel increased. The hydrogel CG01 exhibited the optimum swelling ratio when compared to the equilibrated hydrogel CG02 since it had a higher degree of crosslinking. Additionally, the compressive strength of CG01 was also higher than CG02 in both dry and wet conditions (water and PBS). However, the swelling ability, integrity, and mechanical strength of the CG01 hydrogels improved their biostability. The results indicated that the fabricated cellulose hydrogels without equilibration improved stability in physiological conditions and they could be a suitable hydrogel device for biomedical and tissue engineering applications.

## Figures and Tables

**Table 1:** Total carboxyl content of CG01 and CG02 based on different immersion times in crosslinker solution.

Samples	Total Carboxyl Content (mEq/100 g Hydrogel)						
	1 h	2 h	3 h	6 h	8 h	12 h	24 h
CG01	726	890	1043	1210	1203	1200	1350
CG02	173	146	133	166	183	190	153

**Table 2:** The thermal degradation temperature, % weight loss, and residual content of CG01 and CG02 hydrogels.

Samples	Stages of Decomposition	T (Max) K	% Weight Loss	Residue (%) at 1073 K
CG01	A	472.14	84.48	15.52
	B	581.73		
CG02	A	532.51	71.89	28.11
	B	576.91		

**Table 3:** The mechanical properties of hydrogels at room temperature.

Samples	DRY		WET			
			Distilled Water		PBS	
	Maximum Stress (MPa)	Strain at Max Stress (%)	Maximum Stress (MPa)	Strain at Max Stress (%)	Maximum Stress (MPa)	Strain at Max Stress (%)
CG01	3.55 ± 0.84	70	2.57 ± 0.20	61	1.3 ± 0.09	55
CG02	2.48 ± 0.63	70	2.27 ± 0.98	66	0.25 ± 0.03	42

## Data Availability

Not Available.
